# Individually customized transcranial temporal interference stimulation for focused modulation of deep brain structures: a simulation study with different head models

**DOI:** 10.1038/s41598-020-68660-5

**Published:** 2020-07-16

**Authors:** Sangjun Lee, Chany Lee, Jimin Park, Chang-Hwan Im

**Affiliations:** 1 Department of Biomedical Engineering, Hanyang University, 222 Wangsimni-ro, Seongdong-gu, Seoul, 04763 Republic of Korea; 2 Department of Structure & Function of Neural Network, Korea Brain Research Institute, Daegu, Republic of Korea

**Keywords:** Biophysical models, Biomedical engineering

## Abstract

Temporal interference (TI) stimulation was recently proposed that allows for the stimulation of deep brain structures with neocortical regions being minimally stimulated. For human brain modulation, TI current patterns are known to be considerably affected by the complex structures of the human head, and thus, it is hard to deliver TI current to a specific deep brain region. In this study, we optimized scalp electrode configurations and injection currents that can deliver maximum TI stimulation currents to a specific deep brain region, the head of the right hippocampus in this study, considering the real anatomical head structures of each individual. Three realistic finite element (FE) head models were employed for the optimization of TI stimulation. To generate TI current patterns, two pairs of scalp electrodes were selected, which carry two sinusoidally alternating currents with a small frequency difference. For every possible combination of electrode pairs, optimal injection currents delivering the maximal TI currents to the head of the right hippocampus were determined. The distribution of the optimized TI currents was then compared with that of the unoptimized TI currents and the conventional single frequency alternating current stimulation. Optimization of TI stimulation parameters allows for the delivery of the desired amount of TI current to the target region while effectively reducing the TI currents delivered to cortical regions compared to the other stimulation approaches. Inconsistency of the optimal stimulation conditions suggest that customized stimulation, considering the individual anatomical differences, is necessary for more effective transcranial TI stimulation. Customized transcranial TI stimulation based on the numerical field analysis is expected to enhance the overall effectiveness of noninvasive stimulation of the human deep brain structures.

## Introduction

Transcranial direct current stimulation (tDCS) and transcranial alternating current stimulation (tACS) are noninvasive brain stimulation techniques that can modulate cortical excitability by transmitting weak currents via electrodes attached to scalp surfaces^1,2^. These techniques have been shown to be effective in modulating a variety of brain functions and facilitating neurorehabilitation in Parkinson’s disease, stroke, Alzheimer’s disease, and aphasia^3–5^. It has been known that tDCS facilitates excitability of cortical neurons whereas tACS entrains the endogenous brain oscillations^6,7^. The target brain areas stimulated by tDCS and tACS have mostly been confined to shallow cortical areas. This is because it is generally difficult to selectively stimulate deep brain structures with tDCS or tACS while avoiding unwanted modulations of the neocortical neurons. Although previous studies showed that a certain level of electric field can be delivered to subthalamic regions with tDCS^8^, a computational simulation study has demonstrated that the delivery of much larger electric fields to neocortical regions is inevitable^9^.

Recently, a new noninvasive electrical brain stimulation method called temporal interference (TI) stimulation was proposed to modulate deep brain regions while avoiding unwanted modulation of neocortical neurons^10^. Two temporally interfering sinusoidal electric fields with a small frequency difference (Δ*f*) can form a TI pattern with a frequency equal to the average of the two electric fields and an envelope equal to Δ*f* at specific brain areas. This low frequency component, often referred to as a beat frequency, is delivered only to neurons in a deeper site of the brain. As high frequency electric fields are known not to directly evoke neuronal activities^11^, this novel technique can selectively modulate neurons in the deep brain structures. An animal study with mice also verified that a TI stimulation with two modulation frequencies of 2 kHz and 2.01 kHz could directly activate neurons in the hippocampus with a frequency of 10 Hz, while neurons in the shallow cortical areas were not affected^10^. However, a recent simulation study on transcranial TI stimulation with a realistic human head model showed that it is generally difficult to focalize the TI pattern only around a specific target brain area because of the inhomogeneous conductivity distribution inside the human head^12^. A recent simulation study demonstrated that TI stimulation can deliver electric field large enough to entrain brain oscillations at the frequency of TI envelope in deep brain structures^13^; however, since this study focused only on validating that deep brain areas can be modulated more strongly by using the TI stimulation than the conventional tACS, it did not fully demonstrate that deep brain areas could be modulated selectively with the unwanted modulation of neocortical areas being minimized. Furthermore, this study utilized only a single human head model. Since it is well-known that individual anatomical differences can affect the electric field distributions in the brain during transcranial electrical stimulation^14,15^, feasibility of the optimized TI stimulation needs to be further validated with different head models.

To address the abovementioned issue, in this study, we optimized the parameters of TI stimulation, including electrode configurations and magnitudes of injection currents, with three individual head models, to deliver the maximum TI currents to a designated target region, with TI currents in cortical areas being minimized as much as possible. To evaluate the distribution of TI patterns in the brain, we first solved a quasi-static Laplace equation using the finite element method (FEM) with a realistic head model, enabling the calculation of the electric fields generated by a pair of electrodes, each of them carrying a certain amount of injection current^16,17^. The temporal changes of electric field distributions in the brain were then evaluated by successively conducting the finite element analysis (FEA), followed by Hilbert transform, resulting in the spatial distribution of the TI envelope amplitude at a beat frequency. In this study, the head of the right hippocampus was selected as the target brain structure and three realistic head models generated from individual magnetic resonance imaging (MRI) data were employed for the simulation and optimization. Four electrodes were selected from 61 electrode candidates, and then, optimal injection currents that form the maximum amplitude of TI envelope in hippocampus while reducing the unwanted modulation of the shallow cortical areas were determined. The overall efficacy of the proposed optimization process was compared with that of conventional approaches.

## Methods

### Realistic head models

**Figure 1:**
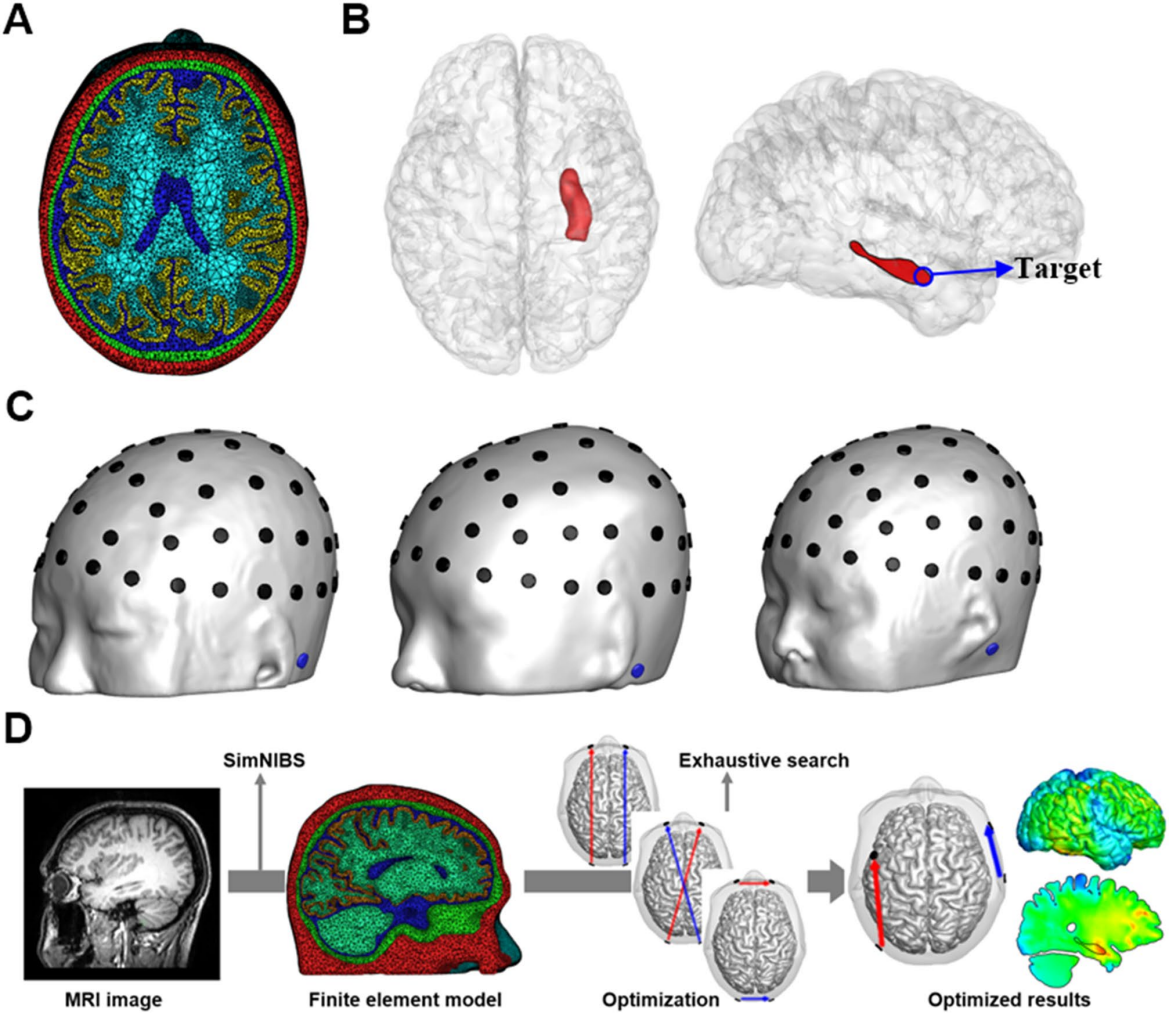
(**a**) Representatively, only the FE head model of sub1 was depicted with five segmented tissues: scalp (red), skull (green), CSF (blue), gray matter (yellow), and white matter (sky blue). (**b**) Illustration of the right hippocampus and the target, the head of the right hippocampus, was marked with blue circle. (**c**) Sixty-one electrode candidates (black) and a return electrode (blue) in the three head models. The location of electrode candidates was decided based on the international 10–10 EEG position. (**d**) The pipeline for the simulation of individualized TI stimulation.

Three realistic finite element (FE) head models were created from three young male subjects (24, 26, and 27 years old) with no clinical history of psychiatric disorders and with no abnormal findings in their magnetic resonance (MR) images. We named their head models H_sub1_, H_sub2_, and H_sub3_, respectively. T1-weighted MR images were acquired from a 3-T MRI scanner with a resolution of 1 × 1 × 1 mm. All subjects were required to provide a written informed consent after they had been informed of the purpose of the experiment. They also agreed the publication of their potentially identifiable head images in an online open-access publication by signing the written informed consent. The experimental protocol was approved by the Institutional Review Board (IRB) Committee of Hanyang University (HYI-17-180-5). All data acquisitions were performed in accordance with the guidelines and regulations set by the IRB of Hanyang University. We used SimNIBS v2.0^18^ for automatic segmentation of tissues including scalp, skull, cerebral spinal fluid (CSF), gray matter, and white matter (see Fig. 1a). The right hippocampus was segmented manually using ITK-SNAP v3.8 (https://www.itksnap.org) and a certain point in the head of the hippocampus was set to the target in this study (see Fig. 1b). Then, an in-house Matlab 2018a (Mathworks, Natick, MA, USA) script was coded to correct segmentation errors and improve the quality of tetrahedral elements by removing isolated nodes and self-intersecting elements. The detailed correction process can be found in a previous literature^19^. The total numbers of elements and nodes of the three head models are listed in Table 1.

**Table 1:** Information of FE models for each head model.

Head models	Number of nodes	Number of elements
H_sub1_	355,405	2,026,831
H_sub2_	430,931	2,513,989
H_sub3_	402,389	2,379,456

### Optimizing electrode configurations and injection currents

The three FE models were used for the determination of optimal electrode configurations and injection currents. Sixty-one electrode candidates were attached according to the international 10–10 EEG electrode system and a reference electrode was assumed to be attached at the left mastoid of each model. The shape of the electrodes was a thin cylinder with a diameter of 1 cm and thickness of 2.5 mm. The electric field intensity vector at each node of the given head model could be evaluated by solving the FEM formulated using the electrostatic Laplace equation given by $-\nabla \cdot \left(\sigma \nabla V\right)=0$, where $\sigma$ represents electrical conductivity and $V$ represents electrical potential. A Dirichlet boundary condition of 1 V was imposed on the upper side of one active electrode and − 1 V was imposed on the upper side of the reference electrode. Then, the electric field and the current density values in the entire analysis domain were scaled by the ratio of the injection current (1 mA) to the computed injection current passing through the bottom of the active electrode^20^. This process was repeated so that the electric field distributions for all 61 cases (61 electrode pairs, each one consisting of one active electrode and the fixed reference electrode) were evaluated and stored in the computer^21^. We assumed that all the tissue compartments are homogeneous and their electrical conductivity values were set to be the same as those used in a previous study^20^. The variation of electrical conductivity values in the high-frequency condition was ignored^12^. The electrical conductivity of the hippocampus was set to be the same as that of gray matter, and the conductivity of the electrodes was assumed to be 1 S/m^22^.

Four electrodes were selected from 61 electrode candidates to form two electrode pairs. Assuming that a specific electrode pair combination was determined, electric field distributions generated by each of the two electrode pairs were calculated as follows: When injection currents of 1 mA and − 1 mA were applied to one electrode pair, the resultant electric field distribution could be readily evaluated by the superposition of two pre-calculated electric field distributions, which are the electric field distribution when currents of 1 mA and − 1 mA are applied to one active electrode and the reference electrode, respectively, and the electric field distribution when currents of 1 mA and − 1 mA are applied to the reference electrode and the other active electrode, respectively (this can be evaluated simply by multiplying − 1 with the pre-calculated electric field distribution)^21^. Then, no current flows in the reference electrode. We can apply the same process to the other electrode pair, thereby resulting in two independent electric field (vector) distributions denoted by ${{\varvec{E}}}_{1}$ and ${{\varvec{E}}}_{2}$. To simulate the TI phenomenon, two sinusoidal currents with different frequencies (${f}_{1}$ and ${f}_{2}$) need to be applied to two electrode pairs, respectively. The electric field at a time sample *t*, denoted by ${\varvec{E}}\left(t\right)$, can be evaluated by the superposition of two electric fields that are sinusoidally alternating with frequencies of ${f}_{1}$ and ${f}_{2}$, as follows:

1 $${\varvec{E}}\left(t\right)=\alpha {{\varvec{E}}}_{1}\mathit{sin}\left(2\pi {f}_{1}t\right)+\beta {{\varvec{E}}}_{2}sin\left(2\pi {f}_{2}t\right),$$

where *α* and *β* are scalar constants that need to be determined. In this study, ${f}_{1}$ and ${f}_{2}$ were assumed to be 2 kHz and 2.01 kHz, respectively, for the generation of an envelope frequency (${f}_{TI}$) of 10 Hz in the target area. After evaluating the time series of the resultant electric field ***E***(*t*), the maximal envelope amplitude *A* of ***E***(*t*) was determined as^12^

2 $$\begin{array}{c}A= 2maxmin(\Vert {{\varvec{E}}}_{1}\Vert \left|cos\theta \right|, \Vert {{\varvec{E}}}_{2}\Vert \left|cos(\theta -\varphi )\right|) ,\\ \theta \end{array}$$

where *φ* represents the angle spanned by ${{\varvec{E}}}_{1}$ and ${{\varvec{E}}}_{2}$, and *θ* denotes the angle between ${{\varvec{E}}}_{1}$ and a unit vector lying on the plane spanned by ${{\varvec{E}}}_{1}$ and ${{\varvec{E}}}_{2}$, based on the fact that the maximal TI envelope only appears when the unit vector lies on the plane spanned by ${{\varvec{E}}}_{1}$ and ${{\varvec{E}}}_{2}$. The angle *θ* ranges from 0 to 2π. The maximal TI envelop amplitude *A* was calculated at every node in the head model. The FEM solver to evaluate electric fields inside the human head was based on that used in the Comets2 toolbox (https://www.cometstool.com) developed by our group, which has been extensively employed by more than 40 research groups all around the world^23–25^ and validated through the comparison with analytic solutions^25^. To deliver maximum TI current with an envelope frequency of ${f}_{TI}$ to the hippocampus while minimizing the TI current formed in neocortical areas, the following objective function to be maximized (hereafter referred to as peak ratio (PR)) was employed with the constraint that *A*_*hippo*_ should be larger than 0.2 V/m according to a previous study reporting that the alternating electric fields larger than 0.2 V/m are sufficient to entrain endogenous brain oscillations in the target region^26,27^:

3 $$ {\text{Objective}}\;{\text{Function}}\;\left( {{\text{PR}}} \right) =  \frac{{A}_{hippo}}{{A}_{cortex}}$$

where *A*_*hippo*_ and *A*_*cortex*_ represent the peak amplitudes of the TI envelope with a frequency of ${f}_{TI}$ at the target point in the head of the right hippocampus and in the cortex, respectively. Note that the peak values were not evaluated only on the surface of the tissues but were evaluated for all nodes inside the tissues. Then, the amplitudes of two sinusoids in (1), *α* and *β*, were determined so that the objective function in (3) could be maximized. We used an exhaustive search approach for the determination of *α* and *β* when the value of *α* was increased from 0.5 to 1.5 mA with a step size of 0.05 mA, while the sum of *α* and *β* was fixed at 2 mA. We applied the above process for all three combinations of electrode pairs that can be made using four selected electrodes, and then selected one electrode pair combination with injection currents of *α* and *β* that maximizes the objective function (3). The same process was repeated for all possible selections of four electrodes among 61 electrode candidates (the number of combinations = _61_C_4_ = 521,855), and then the optimal electrode configuration and injection currents could be determined.

### Comparison with conventional approaches

The effectiveness of our optimization was validated through the comparison with four conventional approaches: (i) unoptimized TI stimulation, (ii) single frequency tACS and (iii) optimized single frequency tACS. For simulating the unoptimized TI stimulation condition, two current-carrying electrode pairs, O1–Fp1 and T6–F8, with alternating frequencies of 2 kHz and 2.01 kHz, respectively, were assumed, with the amplitude of both sinusoids being set to be 1 mA. The two electrode pairs were determined using the exhaustive search so as to make TI amplitude of the target (head of right hippocampus) maximized in the head models with a single homogeneous conductivity value, which is the approach used in the Grossman et al.’s work^10^. Note that the same electrode pairs were selected for all three homogeneous head models by chance. For simulating the single frequency tACS, the same electrode pairs as the optimized ones were employed, while two sinusoidal currents both with a constant frequency of 10 Hz were applied to the two electrode pairs. The amplitudes of the two injection currents were also set to be the same as those of the optimized ones. To determine the optimal tACS condition in the 61-channel electrode configuration, an optimization process used in Guler, et al.^28^ was employed, when the total injection current and the maximum individual injection current were set to 2 mA and 1 mA, respectively.

## Results

**Figure 2:**
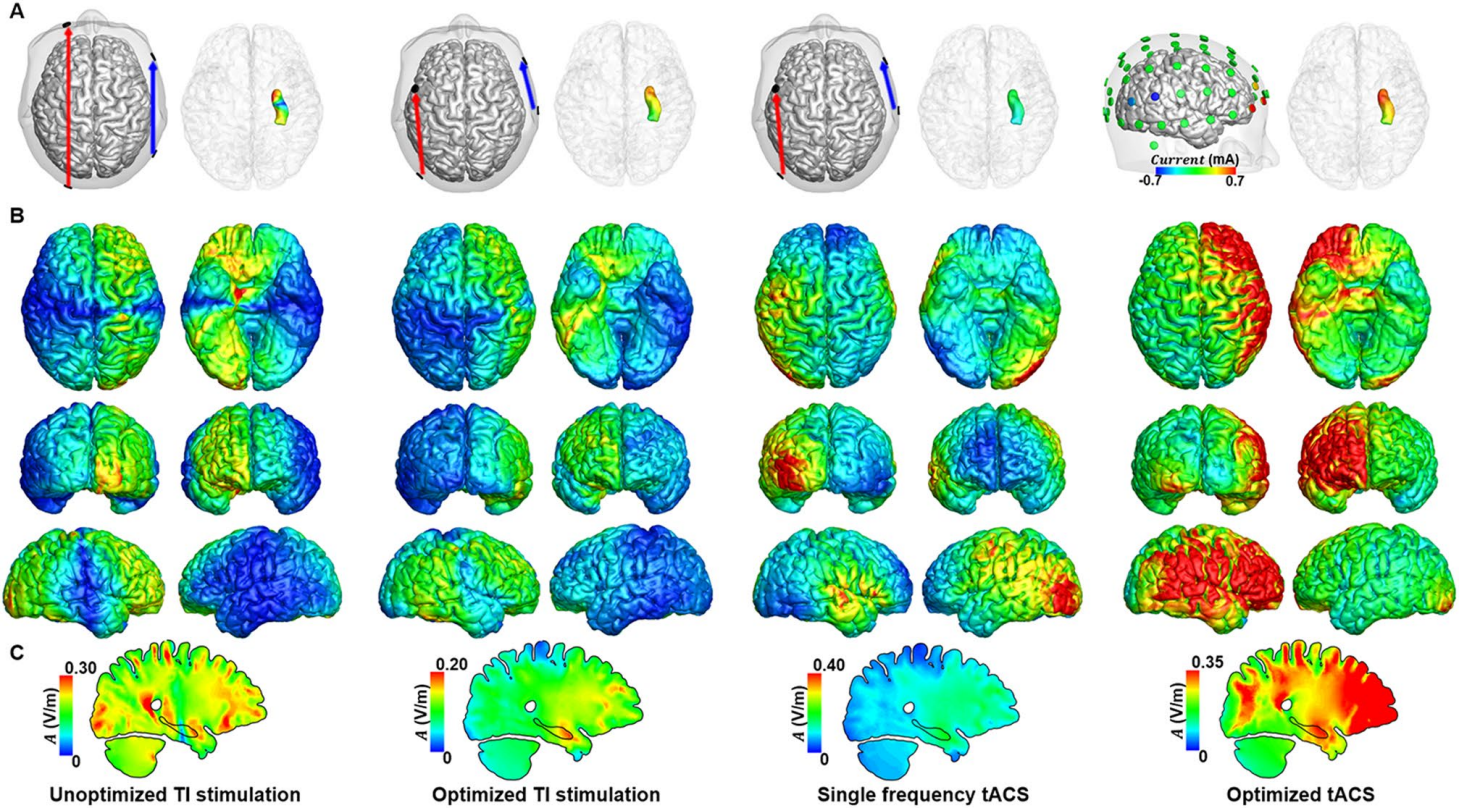
Comparison of the distribution of alternating currents at 10 Hz under four different conditions for H_sub1_. (**a**) Configuration of the two electrode pairs (left) and distribution of the amplitude at 10 Hz in the right hippocampus (right). (**b**) Distribution of the amplitude at 10 Hz in the cortex. (**c**) The medial view of the TI amplitude distribution.

**Figure 3:**
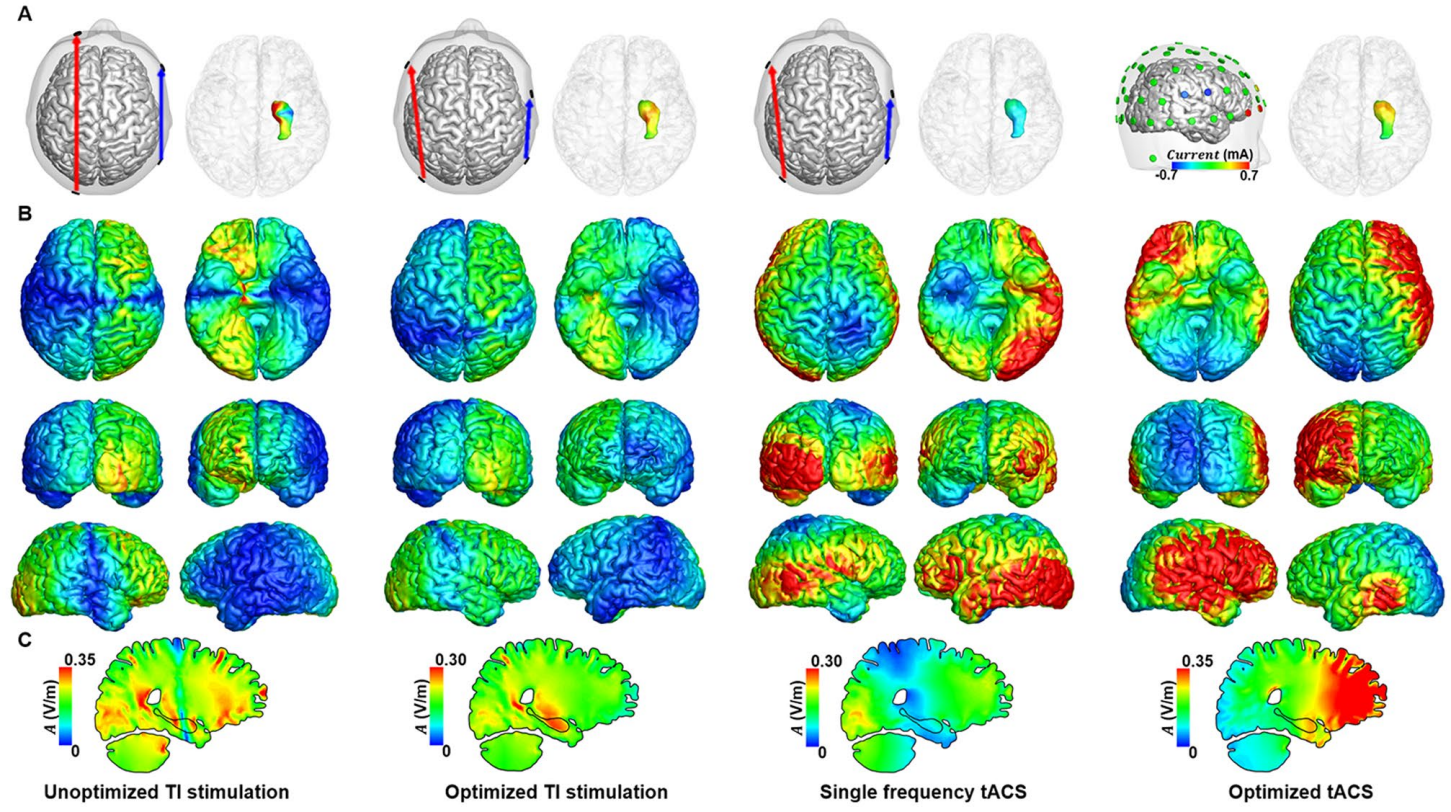
Comparison of the distribution of alternating currents at 10 Hz under four different conditions for H_sub2_. (**a**) Configuration of the two electrode pairs (left) and distribution of the amplitude at 10 Hz in the right hippocampus (right). (**b**) Distribution of the amplitude at 10 Hz in the cortex. (**c**) The medial view of the TI amplitude distribution.

**Figure 4:**
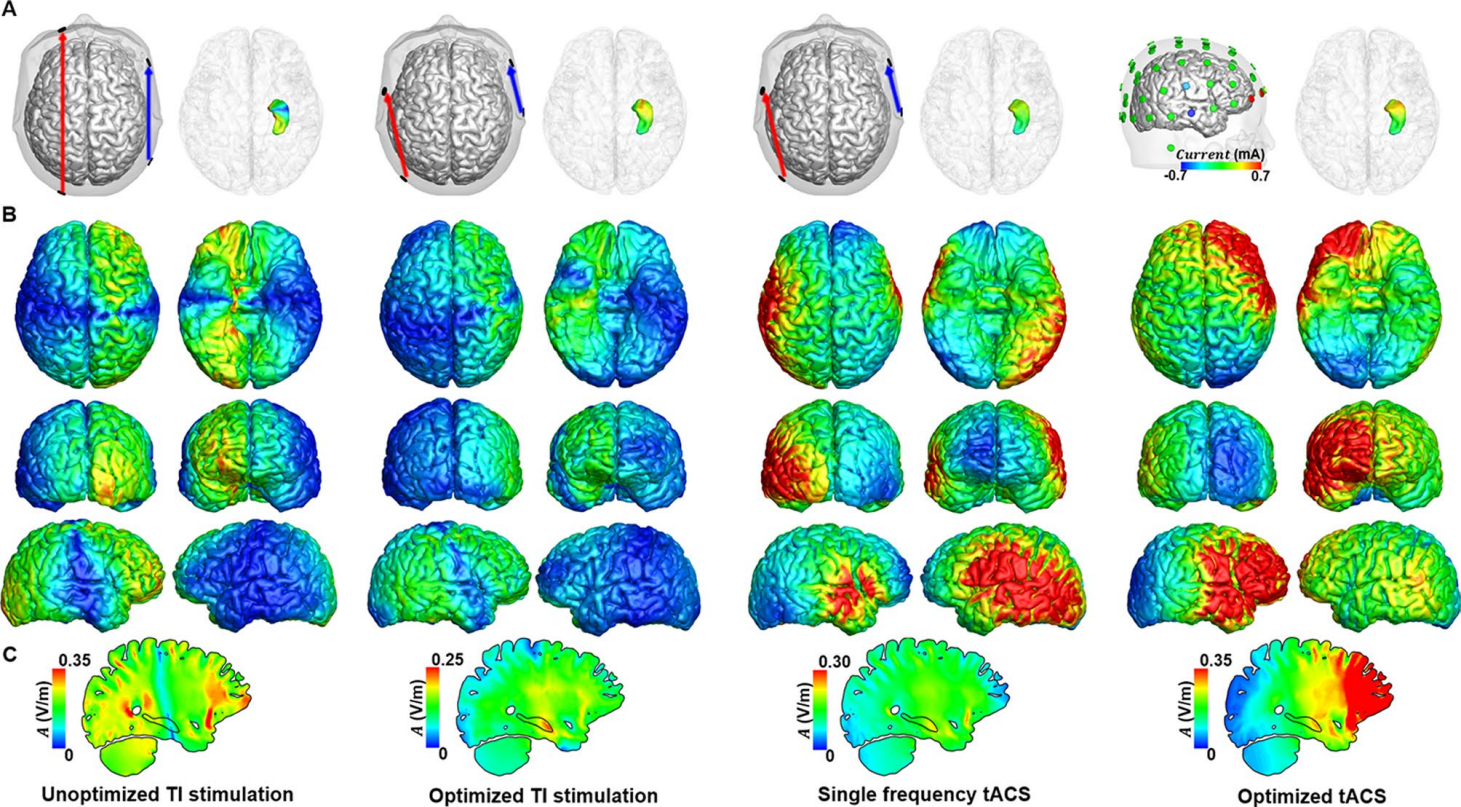
Comparison of the distribution of alternating currents at 10 Hz under four different conditions for H_sub3_. (**a**) Configuration of the two electrode pairs (left) and distribution of the amplitude at 10 Hz in the right hippocampus (right). (**b**) Distribution of the amplitude at 10 Hz in the cortex. (**c**) The medial view of the TI amplitude distribution.

Figures 2, 3 and 4 show the distributions of alternating currents at 10 Hz for four conditions: (i) unoptimized TI stimulation, (ii) optimized TI stimulation, (iii) single frequency tACS, and (iv) optimized tACS. For the head model H_sub1_, the electrode pairs PO7–FC3 and T8–F8 were determined as the optimal electrode configuration (Fig. 2a) when the optimal injection currents were 1.15 mA and 0.85 mA, respectively. For the head models H_sub2_, the electrode pairs PO7-F7 and P8-FC6 (Fig. 3a) with the injection current of 1.25 mA and 0.75 mA were determined as the optimal electrode condition. For the head models H_sub3_, the electrode pairs PO7-FC5 and T8-F8 were determined as the optimal electrode configuration (Fig. 4a) with the optimal injection currents of 1.2 mA and 0.8 mA, respectively. The simulation results demonstrated that the alternating currents delivered to the head of the right hippocampus by transcranial TI stimulation were comparable with those by the conventional tACS. Contrary to the conventional tACS, however, transcranial TI stimulation considerably reduced unwanted modulation of shallow cortical areas, as consistently shown in Figs. 2b, 3b, and 4b. When the current distributions of the optimized TI and unoptimized TI were compared, distinct reduction of modulation currents was observed in various cortical areas including the ventromedial prefrontal cortex, occipital cortex, and prefrontal cortex. Furthermore, the optimized TI stimulation allowed for more focal stimulation of the target, compared to the other conditions (Please see the Figs. 2c, 3c, and 4c). In all cases, the stimulation current flowing through the right hippocampus was the highest when the optimization process was applied.

**Figure 5:**
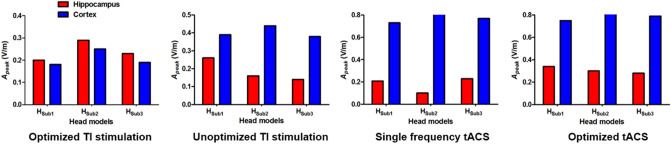
Comparison of peak amplitude at 10 Hz in the head of the right hippocampus and the cortex under four conditions for all head models.

**Figure 6:**
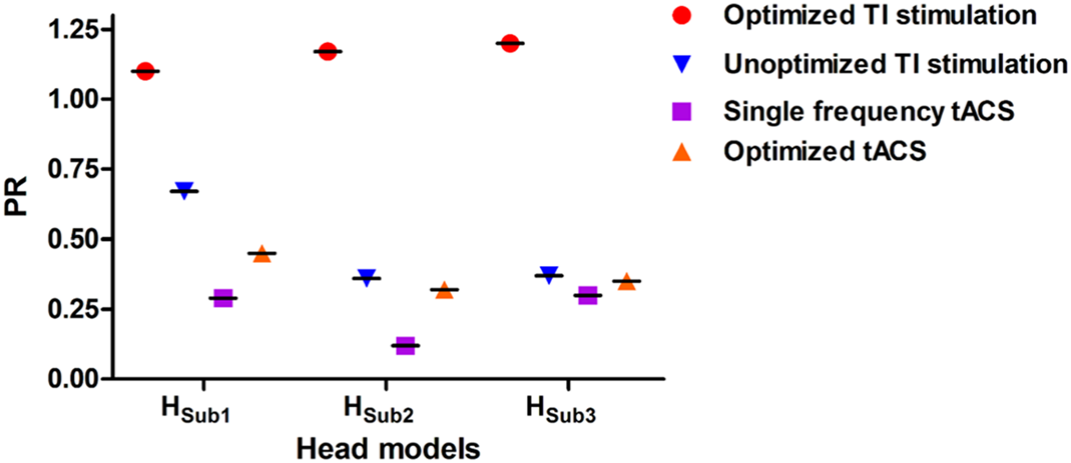
Comparison of PR value representing the ratio of peak amplitude at 10 Hz in the head of the right hippocampus and the cortex under four conditions for all head models.

Figure 5 shows the peak values of 10 Hz currents in the head of the right hippocampus and the cortex. Exact peak values in the head of the right hippocampus and the neocortical regions were listed in Table 2. Further, it is clear that the 10 Hz current delivered to the target was always higher than that delivered to the cortex when optimization was performed. Figure 6 shows the PR values of four different conditions. It can also be seen that the use of TI stimulation considerably increased PR values compared with those obtained by conventional tACS. Further, the optimization process could further increase the PR values, suggesting that the optimization of TI stimulation parameters might allow for the stimulation of the deep brain area while reducing the unwanted modulation of neocortical areas.

**Table 2:** Peak amplitudes of 10 Hz alternating currents in the head of the right hippocampus and neocortical regions and PR values under four different conditions for each head model.

Head models	Optimized TI stimulation			Unoptimized TI stimulation			Single frequency tACS			Optimized tACS		
	Hippo	Cortex	PR	Hippo	Cortex	PR	Hippo	Cortex	PR	Hippo	Cortex	PR
H_sub1_	0.20	0.18	1.1	0.26	0.39	0.67	0.21	0.73	0.29	0.34	0.75	0.45
H_sub2_	0.29	0.25	1.17	0.16	0.44	0.36	0.10	0.85	0.12	0.30	0.94	0.32
H_sub3_	0.23	0.19	1.20	0.14	0.38	0.37	0.23	0.77	0.30	0.28	0.79	0.35

(Unit: V/m).

‘Hippo’ and ‘Cortex’ represent the head of the right hippocampus and the neocortical regions, respectively.

## Discussions

According to a previous study^10^, the TI pattern in the brain can be altered by the variation in the configuration of electrodes and the ratio between two different currents. Based on this, we optimized the locations of electrode pairs and injection currents for individually customized stimulation of deep brain structures (the head of the right hippocampus in this study) while reducing the stimulation intensity of neocortical regions. A numerical analysis based on FEM was employed to compute the three-dimensional distribution of TI currents inside realistic head models. The effectiveness of our optimization was validated through the comparison with the results of unoptimized TI stimulation and single frequency tACS. Our results demonstrated that the optimization of stimulation conditions taking individual head anatomies into account could enable the delivery of the desired amount of stimulation current to a target deep brain area with reduced delivery of stimulation currents to shallow cortical areas compared to the other stimulation conditions. In addition, the difference in the optimal conditions for three head models suggests that customized TI stimulation based on individual electric field analyses might be necessary to enhance the efficacy of TI stimulation.

To further verify the feasibility of the optimization for different target locations, we changed the target location to the tail of the right hippocampus (see the Supplementary Fig. 1 and Supplementary Table 1). The results exhibited PR values greater than 1 for all head models, demonstrating that the individualized TI stimulation may be feasible for other deep brain regions. However, regardless of the target brain areas, unwanted modulation of other deep brain regions was not avoidable. In particular, relatively larger TI currents were formed around the ventricles due to their relatively high electrical conductivity, as also shown in previous TI stimulation studies^10,13^. It seems difficult to prevent the modulation of other deep brain regions with just two electrode pairs. Therefore, it would be an interesting future topic to reduce the unwanted modulation of other deep brain regions by employing more electrode pairs or developing new objective functions.

In our individual optimization results, optimal stimulation conditions were not consistent between head models, which is believed to originate from the difference in individual anatomical structures. Previous studies on tDCS have demonstrated that the electric field distribution inside the head is significantly influenced by the difference in anatomical structures such as skull thickness, CSF thickness, and cortical folding^22,29^. In addition, the location and shape of the right hippocampus might also affect the optimization results in our study. As it has been reported that a slight drift in the location of electrodes produced significant changes in the electric field distribution in the brain^30^, the use of a higher number of electrode candidates might potentially lead to a better optimization result. However, the use of more electrode candidates also increases the computational burden due to the exhaustive searching in the optimization process, and thus, it is necessary to determine the appropriate number of electrode candidates considering the practical usability of our optimization approach. Note that approximately 10 h were required to complete the whole optimization process under a desktop environment (Intel i7-4790 K 4.00 GHz, 32-GB RAM), when 61 electrode candidates were used. Please also note that approximately 8 h were needed to complete the segmentation of brain tissues using SimNIBS.

Among the various brain structures, the head of the right hippocampus was determined as the target region of interest (ROI). Because the hippocampus is closely associated with important brain functions such as the declarative memory and cognitive function^31,32^, researchers have tried to noninvasively stimulate the right hippocampus using transcranial electrical stimulation (tES)^33^. However, even though a certain amount of current can reach the head of the right hippocampus, modulation of the cortical neurons lying on the paths of stimulation currents is inevitable. This makes it difficult to discriminate whether the behavioral changes after the stimulation are solely due to the direct modulation of the hippocampal activity or partly due to the indirect modulation of the hippocampus via functional connections between hippocampal neurons and cortical neurons. Customized TI stimulation based on our optimization approach might address this issue because our approach could effectively reduce unwanted modulation of cortical neurons. Indeed, a previous study that attempted to modulate the temporal cortex^34^ observed modulation of the verbal declarative memory after tACS but could not clearly conclude whether this effect was produced by modulation of the hippocampus or the temporal cortex. Contrary to the single frequency tACS, our simulations showed that the right hippocampus can selectively be modulated with a frequency as low as 10 Hz without a considerable modulation of the temporal cortex.

In our study, high-frequency alternating currents up to 1 mA were injected though small-sized scalp electrodes, the diameter of which was 1.0 cm. Traditionally, it was believed that the use of small-sized electrodes might potentially cause skin burn, skin redness, and pain^35^; however, a recent study reported that injection of 3 mA current through electrodes with a diameter of 0.8 cm did not cause any side effects listed above^36^. In addition, a recent tACS study reported that high-frequency stimulation with 5 kHz alternating current proved to be safe in human subjects^37^. Based on these reports, it is expected that TI stimulation with 1 mA – 2 kHz conditions can be safely applied to human subjects although further animal studies to confirm the safety of TI stimulation would be needed before human trials.

In this study, 0.2 V/m was used as a minimal electric field constraint in the optimization process based on a previous study with animal experiments^27^. However, it should be noted that 0.2 V/m is not a definite threshold value. Indeed, the modulation threshold value is still controversial and may vary individually^38^. For instance, a previous human experimental study showed that the electric field strength ranging from 0.15 to 0.21 V/m can show stimulation effect^39^. In addition, bi-hemispheric phase synchronization of cortical rhythms could be induced even with the electric field strength as low as 0.1 V/m^40^. On the other hand, a previous simulation study with a human head model demonstrated that amplitude-modulated tACS, of which the waveform is similar to that of TI stimulation, needs stronger stimulation intensity than the conventional tACS to expect similar stimulation effect^41^. Despite these studies, the modulation threshold to elicit neural state changes in the human brain with TI stimulation has not been revealed yet. It is also possible that the modulation threshold may be dependent on the brain states and brain tissues, making it difficult to determine a hard threshold value. In our simulations, we assumed that 0.2 V/m is a threshold value that can induce neural state changes regardless of brain tissues just to validate the possibility of optimal stimulation at a target deep brain area; however, this assumption should be updated reflecting new experimental results that will be reported in the future.

On the other hand, the resultant TI envelope amplitude was not high enough to induce action potentials in the neurons, for which electric field of approximately 1 V/m is necessary^42^. We investigated the maximum feasible electric field that can be formed at the target deep brain region. We optimized the stimulation parameters again with a new objective function to maximize peak TI envelope amplitude at the target with a constraint of PR > 1. As shown in the Supplementary Fig. 2 and Supplementary Table 2, the maximum electric field intensity at the target was just 0.38 V/m in the H_sub2_ head model. Note that the transcranial TI stimulation does not aim to directly induce action potential of neurons but modulate states of the neurons in specific brain areas like many other transcranial current stimulation methods^13^.

Compared to other stimulation approaches (unoptimized TI stimulation and conventional tACS), the optimized TI stimulation could successfully increase PR values for all three head models, which can be seen more clearly in Supplementary Fig. 3 that shows the distribution of TI envelope amplitude in the right hippocampus normalized with maximum TI envelope amplitude in the neocortical regions. However, the difference of TI current strengths between the target and neocortical regions was not markedly large even in the optimization results (PR values were still 1.1–1.2). Therefore, in the practical applications, selective stimulation of a specific deep brain tissue might be difficult. We believe that new methods to increase the PR values (e.g., more than 1.5) should be introduced to allow for the selective modulation of deep brain areas. Employment of more electrode pairs might be one of the promising solutions to tackle this issue. In case of tDCS, employment of multiple channels enhanced the focality of the stimulation^21,43^.

A previous study reported that TI stimulation with the optimized electrode conditions allows for delivering TI envelop amplitude larger than the modulation threshold and the optimized TI stimulation enables more focal stimulation of deep brain regions compared to tACS^13^, which is in line with our results. We reinforced the arguments of the previous study with more numbers of head models, and further suggested the need for the individual optimization for improving the effectiveness of TI stimulation. One of the differences between our study and the previous study is that we used the approach of Huang and Parra^12^ whereas the previous study employed the approach of Grossman et al*.*^10^ for the calculation of the maximal TI envelope amplitude in the brain; however, the resultant TI envelop amplitude distributions obtained by both approaches were globally similar to each other^13^. Interestingly, optimized electrode configurations obtained from our study and the previous study^13^ are quite different even though the same target area was assumed. This difference may originate from the different objective functions employed for the optimization. The previous study employed the objective function that minimizes the volume of the whole brain with TI current larger than the modulation threshold. On the other hand, the objective function used in the present study mainly focused on minimizing the stimulation of cortical neurons.

The small number of head models employed in our simulations is a clear limitation of our study. Owing to the limited number of head models, it was difficult to estimate how much PR values vary across the population or evaluate the gender differences. Indeed, a previous study reported that individual local hotspot and strength of electric field in neocortical region were slightly different among head models even though the same regions were targeted with tDCS^29^, suggesting that the difference in individual anatomical structures can lead to inter-subject variability in the electric field distribution. In addition, it was also reported that there can be a gender difference in the strengths of electric fields at targeted regions due to the difference in the anatomical structures^44^. Since the three head models used in our study may not be representative of the general population, employment of more numbers of head models would allow for a better understanding of the TI stimulation as well as statistical comparisons among different stimulation conditions.

We expect that the efficiency of TI stimulation might be further enhanced by employing new approaches in future studies. Firstly, we empirically set the objective function as the ratio of the peak electric fields in the hippocampus and on the cortex. However, as observed in Figs. 2, 3 and 4, the maximum current delivered to the hippocampus was slightly reduced after the optimization. This issue could be addressed by adjusting the weighting of the two quantities (i.e., the peak electric field in the hippocampus and that on the cortex) and building a new objective function. Adding appropriate constraints (e.g., setting a minimum of hippocampal peak current) and solving a constrained optimization problem could be an alternative option. The use of many pairs of electrodes (more than two) might be another solution to enhance the overall performance of TI stimulation. However, it is evident that an increment in the number of electrode pairs would also considerably increase the time required for the optimization process because of the increase in the possible combinations of electrodes. In addition, unlike the present study, works using more electrode pairs would also require more sophisticated optimization algorithms such as the evolution strategy (ES) and genetic algorithm (GA) because of the increase in the number of variables. Nevertheless, the use of more electrode pairs is a promising topic that we want to pursue in our future studies. Secondly, anisotropic conductivity properties of the skull and white matter were not considered because previous studies reported that the difference in electric field between isotropic and anisotropic head models was not markedly large^45–47^. Nevertheless, the anisotropic tissue conductivity will be considered in our future studies to enhance the accuracy of customized TI stimulation. Thirdly, we demonstrated the feasibility of our optimization approach with two targets (the head and the tail of the hippocampus). Testing various deep brain areas would be also an important future research topic. Lastly, the underlying mechanism of the TI effect has not yet been understood well. The fundamental assumption of our stimulation study as well as the previous relevant studies^10,12^ was that the modulatory effect of TI stimulation originates from the low frequency component included in the TI waveform; however, the experimental results presented in the Grossman, et al.^10^ suggest that the actual physical situation might be more complicated. Therefore, it would be a challenging but important research topic to build a more refined computational model better reflecting the actual mechanism of TI stimulation.

## Conclusion

In this study, we demonstrated that individually optimized TI stimulation enables stimulation of the deep brain structure (the head of the right hippocampus in this study) while reducing the amplitude of TI envelope in neocortical regions. Although the difference between TI envelope amplitudes of the target and the neocortical regions was not markedly large, we believe that there is still a room to increase the difference by employing new optimization approaches and introducing multiple pairs of electrodes. Inconsistency of the optimal stimulation conditions among different head models suggested that customized TI stimulation based on the numerical analysis with individual head models might be necessary for effective transcranial TI stimulation. In the future study, the efficacy of the customized TI stimulation needs to be further validated via human trials.

## Supplementary information

Supplementary Information.

## Data Availability

Please contact the corresponding author (ich@hanyang.ac.kr) for data requests.
