# Supplementary material for: Individually customized transcranial temporal interference stimulation for focused modulation of deep brain structures: a simulation study with different head models
Source: Sci Rep. 2020 Jul 16;10:11730. doi: 10.1038/s41598-020-68660-5 (PMC7366675; doi:10.1038/s41598-020-68660-5)
Supplement: Supplementary file 1 — Supplementary Information. [file 41598_2020_68660_MOESM1_ESM.pdf]

Supplementary Information

Individually customized transcranial temporal interference stimulation for focused modulation of deep brain structures: A simulation study with different head models

Sangjun Lee[a], Chany Lee[b], Jimin Park[a], Chang-Hwan Im[a, *]

[a] Department of Biomedical Engineering, Hanyang University, Seoul, Republic of Korea

[b] Department of Structure & Function of Neural Network, Korea Brain Research Institute, Daegu, Republic of Korea

[*]Corresponding Author

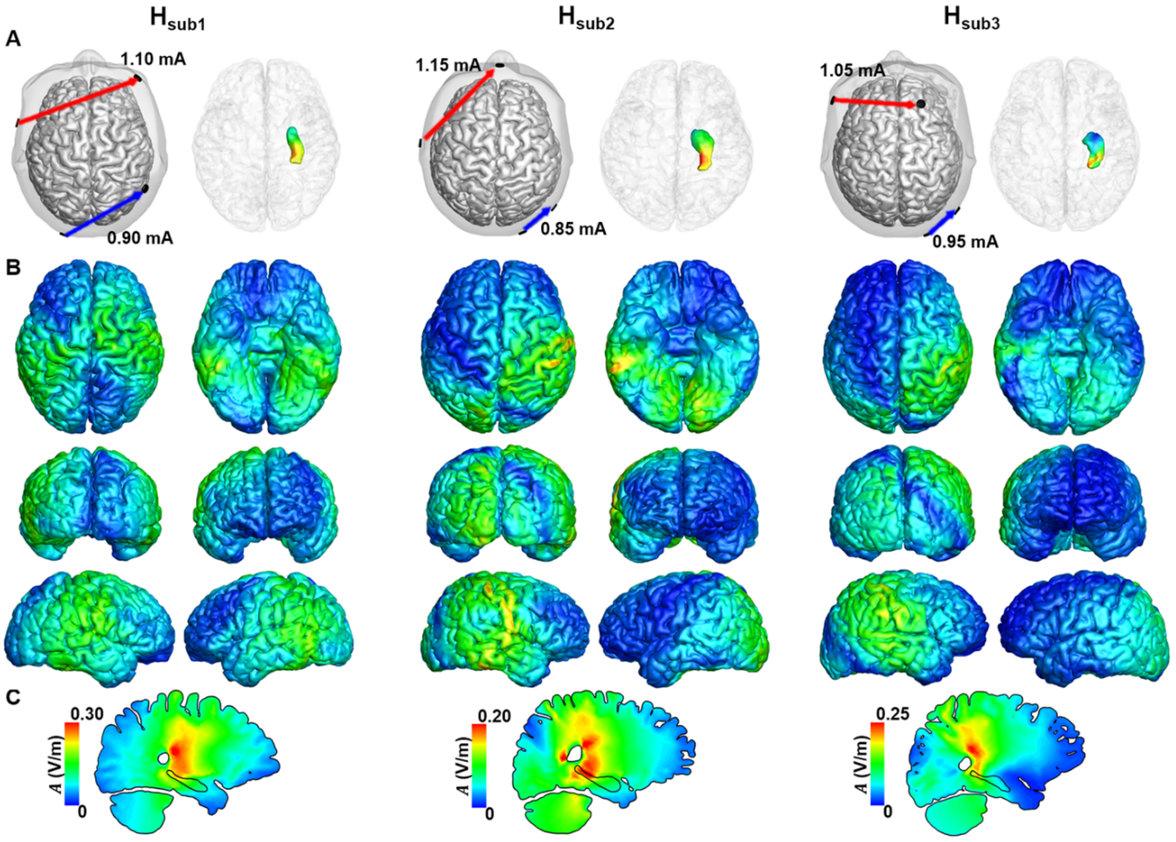

Supplementary Figure 1. Illustration of the electrode configurations and distribution of the TI envelope amplitudes optimized with the objective function set to A_{tail}/A_{cortex}, where A_{tail} represents the peak TI envelope amplitude at the target in the tail of the right hippocampus and A_{cortex} represents the peak TI envelope amplitude in the cortex. (**a**) Configurations of two electrode pairs (FT7-AF8 and O1-CP4 for H_{sub1}, T7-Fpz and O2-P6 for H_{sub2} and F7-F2 and O2-P6 for H_{sub3}) (left) and distribution of TI envelope amplitude in the right hippocampus (right). (**b**) Distribution of the TI envelope amplitude in the cortex. (**c**) The medial view of the TI envelope amplitude distribution in the brain.

Supplementary Table 1. Peak TI envelope amplitudes and PR values optimized with the objective function set to A_{tail}/A_{cortex}. 'A_{tail}', and 'A_{cortex}' represent the peak TI envelope amplitude at the target in the tail of the right hippocampus and neocortical regions, respectively. PR denotes the ratio of A_{tail} to A_{cortex}.

Head models	Optimized TI stimulation		
	A_{tail}	A_{cortex}	PR
H_{sub1}	0.23	0.22	1.05
H_{sub2}	0.21	0.18	1.17
H_{sub3}	0.22	0.19	1.16

(Unit: V/m)

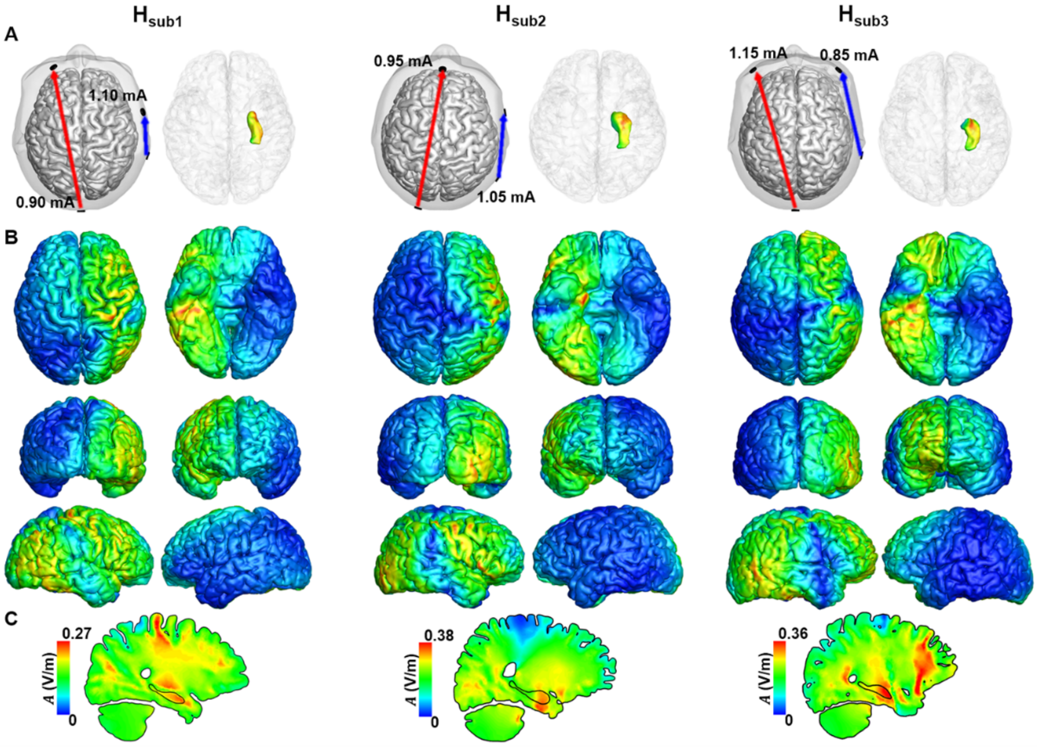

Supplementary Figure 2. Illustration of the electrode configurations and distribution of the TI envelope amplitudes optimized with the objective function set to A_{hippo} with the constraint that PR value should be greater than 1, where A_{hippo} represents the peak TI envelope amplitude at the target of the head of the right hippocampus and PR denote the ratio of A_{hippo} to the peak TI envelope amplitude in neocortical regions. (**a**) Configurations of two electrode pairs (Oz-AF3 and CP8-FC6 for H_{sub1}, O1-AFz and P8-FC8 for H_{sub2} and Oz-AF7 and CP8-AF8 for H_{sub3}) (left) and distribution of TI envelope amplitude in the right hippocampus (right). (**b**) Distribution of the TI envelope amplitude in the cortex. (**c**) The medial view of the TI envelope amplitude distribution in the brain.

Supplementary Table 2. Peak TI envelope amplitudes and PR values optimized with the objective function set to A_{hippo} with the constraint that PR value should be greater than 1. 'A_{hippo}', and 'A_{cortex}' represent the peak TI envelope amplitude at the target in the head of the right hippocampus and neocortical regions, respectively. PR denotes the ratio of A_{hippo} to A_{cortex}.

Head models	Optimized TI stimulation		
	A_{hippo}	A_{cortex}	PR
H_{sub1}	0.27	0.27	1.00
H_{sub2}	0.38	0.38	1.00
H_{sub3}	0.36	0.36	1.00

(Unit: V/m)

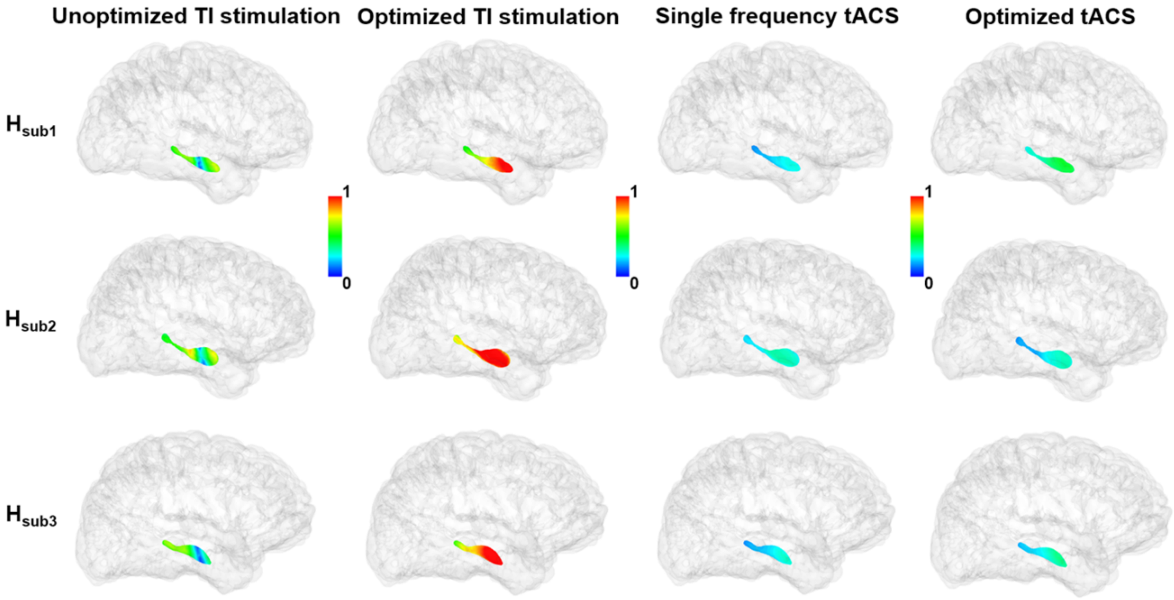

Supplementary Figure 3. Illustration of distributions of the TI envelope amplitude in the right hippocampus normalized with the peak TI envelope amplitude in neocortical area for different stimulation conditions and head models
